# Dural arteriovenous fistula mimicking a stroke: A misdiagnosis of two months

**DOI:** 10.1016/j.radcr.2024.09.043

**Published:** 2024-09-19

**Authors:** Megan Finneran, John Squire, Ajeet Gordhan, Emilio Nardone

**Affiliations:** a Department of Neurosurgery, Carle BroMenn Medical Center, Normal, IL, USA; b Carle Illinois College of Medicine, University of Illinois Urbana-Champaign, Urbana, IL, USA; c Department of Neurointerventional Radiology, Carle BroMenn Medical Center, Normal, IL, USA

**Keywords:** Dural arteriovenous fistula, Mimic, Stroke, Cerebellar

## Abstract

We present a case of a 70-year-old male who presented with left-sided weakness and dysarthria. Cranial imaging was suggestive of a cerebellar infarct and the patient was treated with aspirin and clopidogrel. Two months later a fall prompted further cranial imaging, which was concerning for an intracranial mass with vasogenic edema. Computed tomography angiogram (CTA) was negative for vascular lesion. Ultimately, a DSA revealed a Borden III dAVF between the right occipital artery and the posterior cerebellar vein that was treated with endovascular embolization.

## Introduction

Dural arteriovenous fistulas (dAVF) are an uncommon entity that involve arteriovenous shunting between meningeal arteries and the dural venous sinuses or veins. Digital subtraction angiography (DSA) is the gold standard for diagnosis [1]. Presenting symptoms vary and are largely dependent on location [2]. Imaging findings, particularly on a typical preliminary work-up with head computed tomography (CT) may be nonspecific [3]. Misdiagnosis of dAVF may occur and has been reported to mimic brain tumor tumor [[4], [5], [6]], encephalitis [3], dementia [[7], [8], [9]], venous sinus thrombosis [10], and stroke [[11], [12], [13]].

We report a case of a patient who was misdiagnosed and treated for a cerebellar stroke for several months, then nearly misdiagnosed with a cerebellar mass, prior to a DSA demonstrating the true pathology of a cerebellar dAVF.

## Case presentation

A 70-year-old male with past medical history of coronary artery disease, prostate cancer with prior prostatectomy, colon cancer with prior sigmoid colon resection, hypertension, and previous smoking history presented to an outside emergency department after a fall. He was found to have a right-sided hip fracture and underwent right hip arthroplasty. Upon discharge from the hospital, he was admitted to a skilled nursing facility. On postoperative day ten, he returned to the emergency department for left-sided weakness and dysarthria. Head CT (computed tomography) demonstrated hypodensity within the right cerebellum, causing mass effect on the fourth ventricle. Computed tomography angiogram (CTA) was also performed. Images were unavailable for independent review, but the medical record indicated that the radiologist reported the images were negative for vascular lesion. The report suggested a “probable acute infarction in the right cerebellum.”

**Fig. 1:**
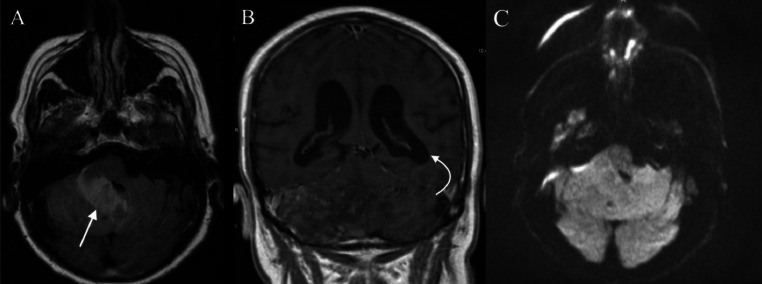
Fluid attenuated inversion recovery (FLAIR) MRI brain from initial hospitalization showed an area of hyperintensity within the right cerebellar hemisphere (straight arrow) on axial imaging (A), causing mass effect on the fourth ventricle. Associated hydrocephalus (curved arrow) was appreciated on coronal imaging (B). Diffusion-weighted imaging (DWI) was negative for diffusion restriction (C).

**Fig. 2:**
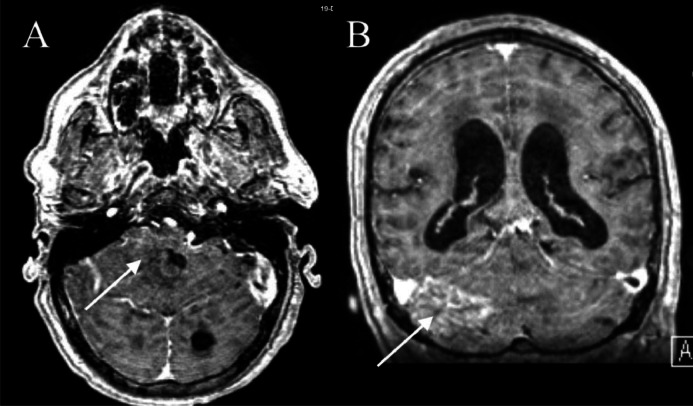
T1-weighted postcontrast MRI brain showed heterogeneous enhancement (arrows) within the area of the precontrast hyperintensity in the right cerebellum, seen on axial (A) and coronal (B) imaging. No discrete lesion was visualized. In retrospect, prominent vessels along the tentorium may have been indicative of an underlying vascular lesion.

An MRI (magnetic resonance image) was subsequently performed that showed an area of hyperintensity on fluid attenuated inversion recovery (FLAIR) MRI, causing mass effect on the fourth ventricle with associated hydrocephalus (Fig. 1). Postcontrast images showed heterogenous enhancement without a discrete lesion (Fig. 2). Records from the outside facility indicate that the patient was believed to have a cerebellar infarct. An EVD (external ventricular drain) was placed and removed 2 days later. The patient was started on aspirin and clopidogrel for a presumed stroke. He was discharged to skilled nursing facility due to persistent left-sided weakness.

**Fig. 3:**
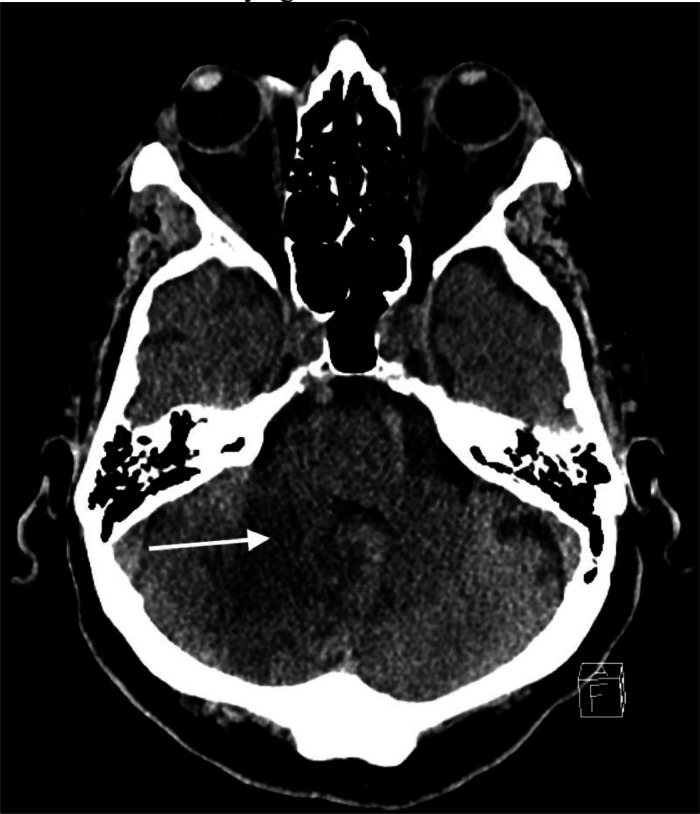
Head CT performed 2 months later, when the patient presented to our institution, demonstrated large area of hypodensity (arrow) within the right cerebellum with associated edema. The patient had originally been diagnosed with a stroke, but the persistent edema raised concern for an underlying lesion.

**Fig. 4:**
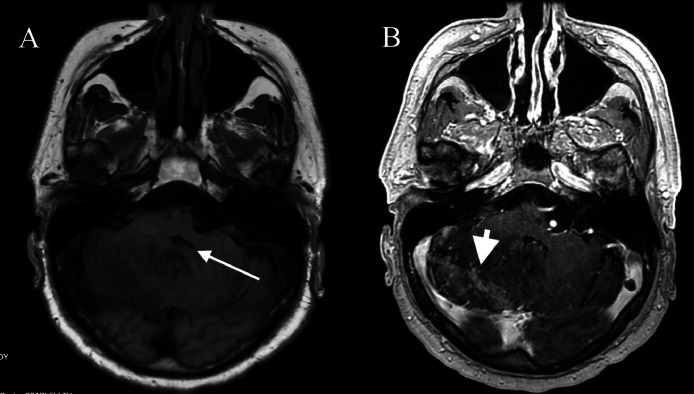
T1-weighted precontrast axial MRI brain (A) showed less marked hyperintensity in the right cerebellum, but worsening edema with almost complete effacement of the fourth ventricle (arrow). T1-weight postcontrast axial MRI brain (B) showed similar patchy enhancement within the area of edema (arrowhead), without obvious mass lesion.

Two months later, the patient had another fall and was taken to our emergency department. He reported left hip pain, but due to the fall he also underwent a head CT. Hip radiograph demonstrated a left hip fracture. Head CT again demonstrated a hypodensity within the right cerebellum with associated edema (Fig. 3). Given the degree of unresolved edema, our team was consulted for possible brain mass. MRI brain showed worsening edema with near effacement of the fourth ventricle (Fig. 4). A CTA was negative for vascular lesion or occlusion. Given his presumed history of stroke, the patient underwent an echocardiogram which showed normal ejection fracture.

At this point the patient was at his reported new neurological baseline according to his wife, with left-sided weakness and dysarthria. Due to the edema, dexamethasone 4 milligrams every 8 hours was initiated. The next day the patient underwent left hip arthroplasty.

Once the fracture was stabilized, a multidisciplinary discussion occurred between the medical team, neurology, radiology, interventional radiology, and neurosurgery. With dexamethasone, the patient had subjective improvement of his symptoms. At this point, the presumed diagnosis of stroke 2 months prior was questioned. Concern was raised that perhaps an underlying mass had been the culprit all along. Neurosurgery was urged to perform a biopsy for further diagnosis. However, given the concern for an underlying vascular lesion, further discussion suggested a digital subtraction angiogram (DSA) as the next step.

**Fig. 5:**
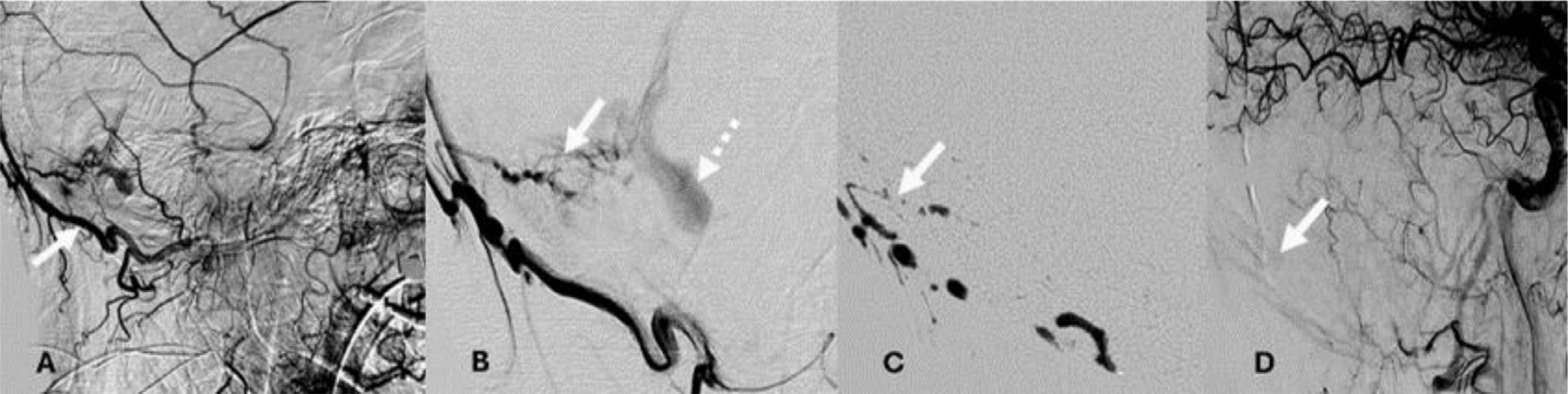
(A) Lateral view digital subtraction angiography (DSA) with right occipital arterial feeder artery injection (white arrow). (B) Micro-catheter injection with demonstration of the dural fistulous connection (white arrow) and recipient vein (dotted arrow). (C) Liquid embolic cast occluding the fistula (white arrow). (D) Post embolization DSA demonstrating disconnection of the fistula.

On postoperative day 2 from the left hip arthroplasty, the patient underwent DSA. The procedure revealed a right posterior, paramedian cerebellar arteriovenous fistula between the branches of the right occipital artery and posterior cerebellar vein (Figs. 5A and B), as well as severe stenosis of the right vertebral artery at its origin.

The patient was continued on dexamethasone 4 milligrams every 8 hours. On postprocedure day eight, he underwent repeat DSA with plans for embolization. Diminutive shunt vascularity related to the distal right occipital artery with slow flow into a venous pouch was identified. The right posterior occipital artery was embolized; complete disconnection was visualized after embolization (Figs. 5C and D).

After embolization, the patient was weaned off dexamethasone. He was discharged on postprocedure day 5 from the embolization to skilled nursing facility. Upon discharge he had persistent left-sided weakness but was otherwise neurologically intact.

## Discussion

Dural AVFs compose approximately 10%-15% of intracranial arteriovenous malformations [11]. They can be difficult to diagnose due to the nonspecific presentation and findings on noninvasive imaging [7]. The exact etiology of dural AVFs remains unclear, but is believed for be associated with venous hypertension [12]. Symptoms vary depending on location, but the hallmark presentation is pulsatile tinnitus due to the increased shunting of blood into the dural venous sinuses [14]. Our patient presented with hemiparesis and dysarthria, symptoms mimicking an ischemic stroke.

Three previous reports have described dAVF mimicking stroke [[11], [12], [13]]. However, all had findings on noninvasive imaging suggestive of dAVF that altered the treatment course. None of them were misdiagnosed and anticoagulated for several months, as our patient was prior to presenting to our institution.

One case described an 80-year-old female who presented with “stroke-like symptoms” of confusion, hemiparesis, and aphasia. However, imaging revealed a vascular malformation and the patient was not treated as an ischemic stroke [11].

Another report consistent with stroke on presentation involved a 76-year-old man who presents with aphasia [12]. Initial head CT was negative for hemorrhage and the patient was given intravenous (IV) alteplase for thrombolysis. After initiation of alteplase, a CTA was performed that showed hyperperfusion in the left temporal lobe, consistent with epileptic activity. Despite halting the infusion, the patient developed an intraparenchymal hemorrhage 12 hours later. Seven days later, further investigation of the CTA showed early filling of the left transverse sinus. The patient then underwent a DSA, which showed a dAVF.

A similar presentation highlighted a 66-year-old female who presented in Singapore with receptive dysphasia [13]. Head CT was negative and the patient was treated with IV alteplase. CTA was performed after the bolus, which revealed a dAVF. Despite administration of fresh frozen plasma, the patient deteriorated rapidly. Head CT showed a large intraparenchymal hematoma. The patient expired shortly later.

Head CT and CTA for all suspected ischemic stroke patients has been supported in the literature [15]. However, no noninvasive imaging can provide 100% sensitivity to rule out dAVF. Noninvasive imaging findings may include cortical venous reflux, indicated by flow voids or abnormal dilatation or early enhancement of vessels [16]. One study of 108 patients found the diagnostic sensitivity of CTA in diagnosing dAVF to be 62 to 96%, while sensitivity of magnetic resonance angiography (MRA) was 58%-83% [17]. Silent MRA is a developing noncontrast enhanced method of angiography that has been proposed to better visualize cerebrovascular lesions in a noninvasive fashion [18]. Silent MRA has shown to be superior for detection of dAVF when compared to time-of-flight MRA, but it makes scanning time ten minutes longer [19].

Although rare, dAVFs should remain in the differential diagnosis of brain conditions that present atypically or do not resolve with treatment as these patients are at high risk of life-threatening complications of true strokes or hemorrhage [20]. This case presentation, and others similar to it, should urge all members of the multidisciplinary team caring for patients who present with neurological symptoms to include dural arteriovenous fistula in the differential. Specifically, an intracranial lesion with nonspecific edema should raise suspicion for dAVF, as venous congestion is common [12]. DSA remains the gold standard and should be heavily considered in such cases, even in the setting of negative findings in noninvasive imaging modalities [3].

## Conclusion

It is important to keep dural arteriovenous fistulas in mind when creating a differential diagnosis for a patient who presents with neurological findings, even in the absence of vascular abnormality on noninvasive imaging. Special consideration should be made when an abnormal pattern of edema is seen, which may be indicative of venous congestion. A low threshold should be maintained for the consideration of digital subtraction angiogram.

## Patient consent

Written, informed consent for publication of the case was obtained from the patient.
